# A Rare Novel Deletion of the Tyrosine Hydroxylase Gene in Parkinson Disease

**DOI:** 10.1002/humu.21351

**Published:** 2010-10

**Authors:** Güney Bademci, Todd L Edwards, Andre L Torres, William K Scott, Stephan Züchner, Eden R Martin, Jeffery M Vance, Liyong Wang

**Affiliations:** 1 University of Miami, Miller School of Medicine, John P. Hussman Institute for Human Genomics MiamiFL 33136, USA; 2 Institute of Medicine and Public Health, Division of Epidemiology, Department of Medicine, Vanderbilt Epidemiology Center, Vanderbilt University School of MedicineNashville, TN 37203, USA; 3 University of Miami, Miller School of Medicine, Dr. John T. Macdonald Foundation Department of Human Genetics MiamiFL 33136, USA

**Keywords:** Parkinson disease, *TH*, deletion, CNV, rare variants

## Abstract

Tyrosine hydroxylase (TH) enzyme is a rate limiting enzyme in dopamine biosynthesis. Missense mutation in both alleles of the *TH* gene is known to cause dopamine-related phenotypes, including dystonia and infantile Parkinsonism. However, it is not clear if single allele mutation in *TH* modifies the susceptibility to the adult form of Parkinson disease (PD). We reported a novel deletion of entire *TH* gene in an adult with PD. The deletion was first identified by copy number variation (CNV) analysis in a genome-wide association study using Illumina Infinium BeadChips. After screening 635 cases and 642 controls, the deletion was found in one PD case but not in any control. The deletion was confirmed by multiple quantitative PCR (qPCR) assays. There is no additional exonic single nucleotide variant in the one copy of *TH* gene of the patient. The patient has an age-at-onset of 54 years, no evidence for dystonia, and was responsive to L-DOPA. This case supports the importance of the *TH* gene in PD pathogenesis and raises more attention to rare variants in *candidate genes* being a risk factor for Parkinson disease. © 2010 Wiley-Liss, Inc.

## INTRODUCTION

Parkinson disease (PD) is characterized by a progressive loss of dopaminergic neurons in the substantia nigra. The rate limiting step in dopamine biosynthesis is catalyzed by the enzyme encoded by the tyrosine hydroxylase (*TH*; MIM# 191290 ) gene (Haavik and Toska., 1998). Consistent with its essential role in dopamine homeostasis, homozygous missense mutations in *TH* have been associated with dopamine-related phenotypes, such as Segawa's syndrome, L-DOPA responsive infantile parkinsonism, and L-DOPA responsive dystonia (DRD) (Furukawa et al., 2001; Hertz et al., 2006). However, it is not clear whether a single allele mutation in *TH* would modify an individual's susceptibility to PD.

## CASE

The patient had an age-at-onset of PD at 54 years, presented clinically with difficulty in the dexterity of the right hand and a typical asymmetric presentation. The patient's symptoms were responsive to L-DOPA. Examination at the time of ascertainment revealed a Hoehn-Yahr rating of 2, Schwab and England level of 90%, and UPDRS part III motor score of 23 when tested in an on state. There was no evidence of dystonia by exam or by history. Both parents of the patient were reported to have been without any clinical symptoms of PD, well into their 70's and 80's. Blood was collected from the patient when she was 59 years old and no other family members are available for DNA testing.

## MATERIALS AND METHODS

### Samples

635 PD cases and 255 PD controls were collected by the Morris K. Udall Parkinson Disease Research Center of Excellence (PDRCE) at Duke University and then at the University of Miami (J.M. Vance, PI), and the 13 centers of the Parkinson Disease Genetics Collaboration. All individuals with PD were examined by a board-certified neurologist. A neurological exam and standard clinical evaluation was performed. An individual was classified as affected if he/she exhibited at least two cardinal symptoms of PD, e.g. bradykinesia, resting tremor, and rigidity and no other causes of Parkinsonism or atypical clinical features. Unaffected individuals had no symptoms of PD based upon physical examination (Edwards et al., 2010). In addition to the 255 PD controls, 387 cognitively- normal controls with no PD symptoms (by self-reported symptom questionnaire) (Rocca et al., 1998) were collected by an ongoing genetic study on late-onset Alzheimer disease (LOAD) (Beecham et al., 2009).

### Methods

For the SNP array based copy number variation (CNV) analysis, genotypes for 635 PD cases and 255 PD controls were generated using the Illumina Infinium 610-quad BeadChip (Illumina) and the Illumina Infinium II assay protocol (Gunderson et al., 2005; Edwards et al., 2010). 223 cognitively-normal controls from the LOAD study were genotyped using the Illumina HumanHap 550 BeadChip and 164 cognitively-normal controls from a second LOAD study were genotyped using the Illumina1M-Duo Infinium HD BeadChip. Genotypes were determined using Illumina BeadStudio Genotyping Module version 3.2.33. CNV events were determined using the PennCNV software, which uses both the Log_2_R ratio and b allele frequency to determine CNV (Wang et al., 2007).

For the qPCR analysis, genomic DNA for the PD patient with *TH* deletion and two DNA samples that have two copies in the *TH* region, as indicated by the SNP array analysis, were evaluated for quantity and quality via an ND-8000 8-Sample Spectrophotometer (NanoDrop®) and agarose gel electrophoresis. Only intact genomic DNA was used to for qPCR analysis. Taqman® copy number assays were custom designed and manufactured by Applied Biosystems (ABI). Conditions for the qPCR reactions were as follows: 1 μl CNV probes (20x, FAM labeled), 1 μl RNaseP probe mix (20x, VIC labeled), 10 μl TaqMan® Universal PCR Master Mix (2×), 1.5 μl genomic DNA and 6.5 μl of water were mixed with a final reaction volume to 20 μl. Reactions were held at 95 °C for 10 min and then cycled 40 times through 95 °C for 15 s and 60 °C for 1 min. Samples were run on the ABI7900HT Fast real-time PCR System and analyzed using Sequence Detection Software version 2.3 (Applied Biosystems). CopyCaller® software was used for analyzing qPCR data using a delta delta Ct algorithm (Applied Biosystems). As recommended by the manufacturer, the RnaseP gene was used as an endogenous control to calculate the delta Ct. Calibration with a control sample with two copies of *TH* was used to calculate delta delta Ct values and assign copy numbers.

For the sequencing analysis, primers of all 14 exons of *TH* were designed using Primer3® software. DNA sequencing analysis of all 14 exons of the *TH* gene was performed with BigDye® Terminator Cycle sequencing Kit and PRISM® 3130 sequencer following manufacturer's instructions (Applied Biosystems).

## RESULTS

**Figure 1:**
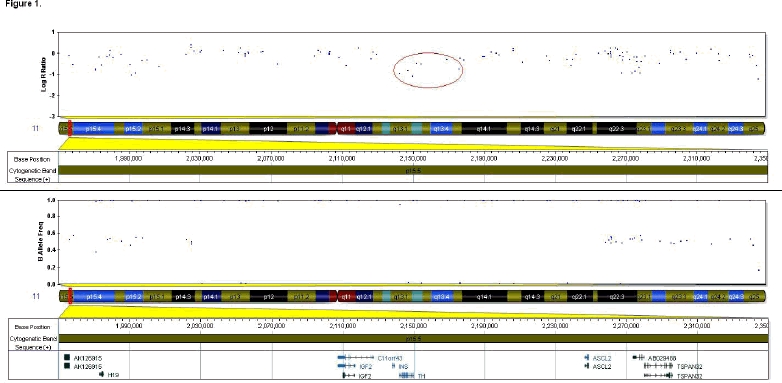
A heterozygous deletion over *TH* identified by SNP array. SNP array data were processed using Illumina BeadStudio software using NCBI36/Hg18 as the reference genome. The region shown is indicated by the red box on chromosome 11p15.4. A hemizygous deletion over *TH* is evident by lower log R ratio and a loss of heterozygotes in the B allele frequency. The seven SNPs that defined the deletion are indicated by the red circle.

**Figure 2:**
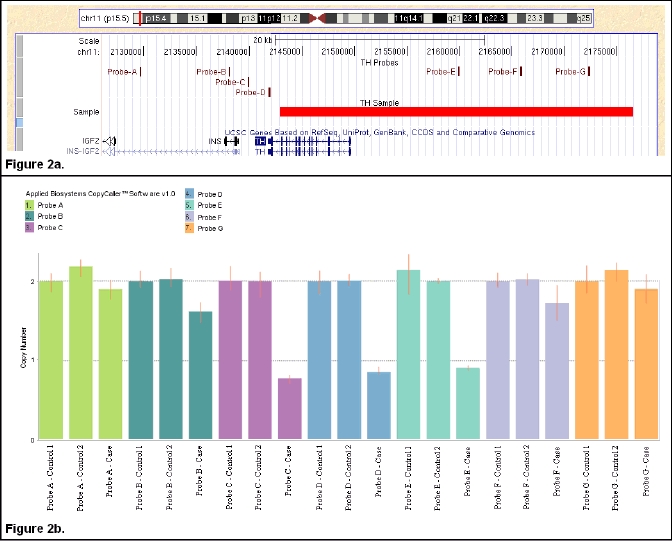
qPCR confirmation of the deletion over *TH*. The hemizygous deletion (red horizontal bar) delimited by PennCNV and the custom designed CNV probes A-G, shown above, displayed on UCSC (Kent et al., 2002) genome browser (NCBI36/Hg18, Figure 2a); Taqman® CNV assay results in the patient and two controls samples (Figure 2b).

The deletion was first found in one subject during a genome-wide CNV analysis using SNP array (Figure 1). In total, 635 PD cases and 642 unrelated controls were included in the analysis. A 34 kilobase deletion over the *TH* gene was found in one PD patient but not in any controls. The deletion is defined by seven SNPs from the Illumina 610-quad Bead chip from rs2070762 to rs3922756 (Figure 2a, red bar).

To validate and fine map the deletion delimited by the SNP array analysis, seven Taqman® copy number assays were designed to cover *TH* and surround regions: CNVA-G, proximal to distal (Figure 2a). For the patient, CNVC, CNVD and CNVE demonstrated one copy deletion while CNVA, CNVB, CNVF and CNVG showed two copies, suggesting that the deletion starts between CNVB and CNVC, ends between CNVE and CNVF on chromosome 11 (Figure 2). *TH* is the only gene residing in the deleted region. For control samples, all copy number assays showed two copies as expected. Therefore, the targeted Taqman® copy number assays confirmed the heterozygous deletion of the whole *TH* gene in the patient and narrowed the genome-wide CNV analysis defined deletion.

All 14 exons of the non-deleted copy of *TH* gene were resequenced in the patient. No single-nucleotide variant was found on the remaining allele.

## DISCUSSION

Our finding suggests that haploinsufficiency of *TH* could increase PD risk. Several lines of evidence support this hypothesis. 1) A carrier parent of a child with DRD, with a *TH* heterozygous missense mutation (296delT), was noted to have stress induced stiffness (Furukawa et al., 2001). 2) A heterozygous missense mutation (A6T) in *TH* was reported in an early-onset PD patient (age-at-onset of 36). The patient also has a heterozygous duplication of exon 11 in the parkin gene. An additive effect of the two mutations may account for developing PD at an early age-at-onset in this patient (Hertz et al., 2006). 3) Animal studies have shown that *TH* enzyme activity decreases by age. This age-associated change could introduce stress to dopaminergic cells and contribute to the risk of PD posed by aging (De La Cruz et al., 1996). It is intuitive to propose that haploinsufficiency of the *TH* gene leads to a lower level of *TH* activity and thus increases PD risk. However, accurate measurement of *TH* activity in the brain requires cerebral spinal fluid analysis (Zafeiriou et al., 2009) and this could not be pursued in this patient.

Our study is an example of how a rare genetic variant could contribute to PD risk. The *TH* deletion appears to be very rare: one case with *TH* deletion was found after screening 635 PD cases in our study. Liu et al. has screened *TH* gene for CNV using Multiplex Ligation-dependent Probe Amplification assays in 16 DRD patients. No deletion was reported, which might be due to the small sample size (Liu et al., 2010). This highlights the difficulty in studying rare variants: it often requires screening large number of samples.

It has been suggested that a large portion of the genetic contribution to complex diseases, like PD, may be due to rare variants (Bodmer and Bonilla., 2008). Each rare variant is expected to be found in a small number of individuals. To definitively demonstrate the association between rare variants and a disease can be difficult, often requiring clustering of multiple rare variants in one gene (Bodmer and Bonilla., 2008), or by conducting a functional study on the rare variant and the gene. In this case, the implication of *TH* in PD is well acknowledged. Due to the limited resolution of the current methods for CNV study, it is possible that smaller deletions in *TH* exist in PD patients but were not identified in our study. Recent leaps in sequencing technology should help answer this question, and catalogue other rare variants in PD.
